# Bioinformatics Analysis Identifies Potential Ferroptosis Key Gene in Type 2 Diabetic Islet Dysfunction

**DOI:** 10.3389/fendo.2022.904312

**Published:** 2022-07-11

**Authors:** Haowen Ye, Ruxin Wang, Jinjing Wei, Ying Wang, Xiaofang Zhang, Lihong Wang

**Affiliations:** ^1^ Department of Endocrinology and Metabolism, First Affiliated Hospital of Jinan University, Guangzhou, China; ^2^ Clinical Experimental Center, First Affiliated Hospital of Jinan University, Guangzhou, China

**Keywords:** T2DM, islet β cell, ferroptosis, bioinformatic, WGCNA

## Abstract

**Background:**

Islet β cells dysfunction (IBCD) is a cortical component in pathogenesis of type 2 diabetic mellitus (T2DM). However, the relationship of ferroptosis and IBCD remains unknown. This study was aimed to screen potential ferroptosis key genes to reveal latent physiological and pathological process of IBCD in T2DM.

**Methods:**

Firstly, T2DM key genes were screened by combining with differentially expressed genes (DEGs) analysis and WGCNA. Then, ferroptosis-related genes (FRGs) in IBCD of T2DM were identified by taking the intersection between T2DM key genes and FRGs. Finally, T2DM-FRGs were validated in another T2DM dataset as well as islet single-cell RNA sequencing dataset and the miRNA regulated T2DM-FRG was predicted by using four miRNA databases.

**Results:**

89 T2DM key genes were identified between DEGs and WGCNA. Then, 3 T2DM-FRGs were screened by taking the intersection of T2DM key genes and FRGs, namely *ITGA6*, *MGST1* and *ENO2*. At last, *MGST1* were validated as the T2DM-FRG in another T2DM islet issues dataset and islet single-cell RNA sequencing dataset.

**Conclusion:**

*MGST1* may be the potential ferroptosis key gene of IBCD in T2DM.

## Introduction

According to International Diabetes Federation (IDF) reports, there are approximately 536.6 million adult diabetic peoples worldwide and almost half of patients were unaware of their diabetes status (1, 2). Some 90% of diabetic individuals have type 2 diabetes mellitus (T2DM), which is currently the most common type of diabetes mellitus and increases the burden on the state. These patients have higher risks of cardiovascular damage, nerve damage, bacterial infection and other complications (3). The typical features of T2DM are glucotoxicity, lipotoxicity, insulin resistance and islet β cells dysfunction (IBCD) (4). IBCD occurs in progressive stage of T2DM and influences the prognosis of T2DM. Understanding the clear mechanism of IBCD can help us to early identify IBCD and perform targeted therapy in T2DM. At present, studies show that the mechanisms of IBCD in T2DM include mitochondrial dysfunction, inflammation, autophagy, oxidative stress (5). But the specific pathogenesis of IBCD in T2DM is still not completely clear and failure to discerned early.

Ferroptosis, a form of nonapoptotic regulated cell death (RCD), is caused by the lipid peroxide accumulation dependent on iron and antioxidant system dysfunction (6). It is worth noting that islet β cells express low level of antioxidant enzymes such as superoxide dismutase (SOD), glutathione (GSH) peroxidase and catalase, so they are susceptible to suffer from oxidative stress (7). Interestingly, lipid peroxide and iron accumulation were observed in type 2 diabetic islet β cells (8, 9). In addition, the islet β cells significantly died and its capacity of glucose-stimulated insulin secretion reduced, when they were treated with ferroptosis inducer erastin *in vitro (* 10). These adverse effects improved in ferroptosis inhibitors Ferrostatin-1- and desferrioxamine-treated islets β cells (10). Thus, IBCD in T2DM probably related to ferroptosis. Probing the underlying molecular mechanism between IBCD in T2DM and ferroptosis may be a breakthrough for its early diagnosis and treatment.

Here, based on the above researches, we screened the key genes by differential expression genes (DEGs) analysis and weighted gene co-expression network analysis (WGCNA) (11) in order to explore the effect of ferroptosis on type 2 diabetic IBCD. These T2DM key genes were intersected with the ferroptosis-related genes (FRGs) to acquire T2DM-FRGs in IBCD. We also validated the T2DM-FRGs using another T2DM islet issues dataset as well as islet single-cell RNA sequencing dataset and predicted microRNAs (miRNAs) regulated T2DM-FRG.

## Materials and Methods

### Datasets

Four T2DM-related microarray datasets GSE38642 (platform: GPL6244) (12), GSE41762 (platform: GPL6244) (13), GSE76894 (platform: GPL570) (14) and GSE76895 (platform: GPL570) (15) were downloaded by “GEOquery” R package from the Gene Expression Omnibus (GEO, https://www.ncbi.nlm.nih.gov/geo/). The islet single-cell RNA sequencing dataset E-MTAB-5061 was downloaded from ArrayExpress (https://www.ebi.ac.uk/arrayexpress/) (16). GSE38642, GSE41762 and GSE76894 were used to elected DEGs. GSE76895 and E-MTAB-5061 were used to verify T2DM-FRGs. The dataset GSE38642 contains 9 T2DM human islet samples and 54 normal human islet samples. The dataset GSE41762 contains 20 T2DM human islet samples and 57 normal human islet samples, while dataset GSE76894 contains 19 T2DM human islet samples and 84 normal human islet samples. Dataset GSE76895 contains 36 T2DM human islet samples and 32 normal human islet samples. Dataset E-MTAB-5061 included 99 β cells samples in 4 T2DM and 171 β cells samples in 6 normal patients. We found 599 ferroptosis-related genes from ferroptosis database (FerrDb, http://www.zhounan.org/ferrdb/legacy/index.html), Genecards database (https://www.genecards.org/) and two new researches about FRGs (17, 18).

### Differentially Expressed Genes Analysis

DEGs were identified by “limma” R package in R software. We considered DEGs as *p*-value <0.05 and |log_2_fold change (FC)| >log_2_1.2. DEGs were visualized by the “volcano” and “heatmap” R packages. The common up-regulated and down-regulated DEGs between GSE38642, GSE41762 and GSE76894 were identified and visualized by Venn diagram in online tool Hiplot (https://hiplot.com.cn/).

### Functional Enrichment Analysis

Online tool DAVID (https://david.ncifcrf.gov/) (19) was used to perform the gene ontology (GO) (20) and kyoto encyclopedia of genes and genomes (KEGG) analyses (21). *p*-value <0.05 was considered as the threshold. GO analysis including biological process (BP) and molecular function (MF). The records of GO and KEGG analysis were visualized as chord chart and bubble diagrams by “ggplot2” R package respectively.

### Weighted Gene Co-Expression Network Analysis (WGCNA)

WGCNA is a well-established method for analyzing the relationship between diseases phenotype and genes (11). The dataset GSE76894, which had a large sample size, was chosen to find key genes related type 2 diabetic phenotype by “WGCNA” R package. A scale-free network was constructed by calculating different power value (range from 1 to 30), and the suitable power value was chosen when scale free *R* ^2^ was 0.85. Subsequently, we performed the cluster analysis to recognize the highly similar modules and the min module size set as 30. The connection between modules and traits were calculated to identify the key trait-module genes. In the module-trait analysis, the module key genes were chosen with module membership (MM) >0.8 and gene significance (GS) >0.2.

### Statistical Analysis

GraphPad Prism 8.0 was used to perform statistical analysis and drawing. We selected unpair t-test as the statistical method. *p*-value <0.05 was considered as statistically significant. Data were presented as mean ± standard deviation (SD).

### miRNA Prediction

mRNA-miRNA was predicted by Targetscan (http://www.targetscan.org/vert_71/), TarBase v.8 (http://www.microrna.gr/tarbase), miRDB (http://mirdb.org/) and ENCORI (https://starbase.sysu.edu.cn/). The miRNAs of interation in databases above were as the predicted miRNA that may regulate T2DM-FRG. The miRNA-gene network was visualized by cytoscape software.

## Results

### Identification of DEGs Between T2DM and Normal Islet Issues

**Figure 1:**
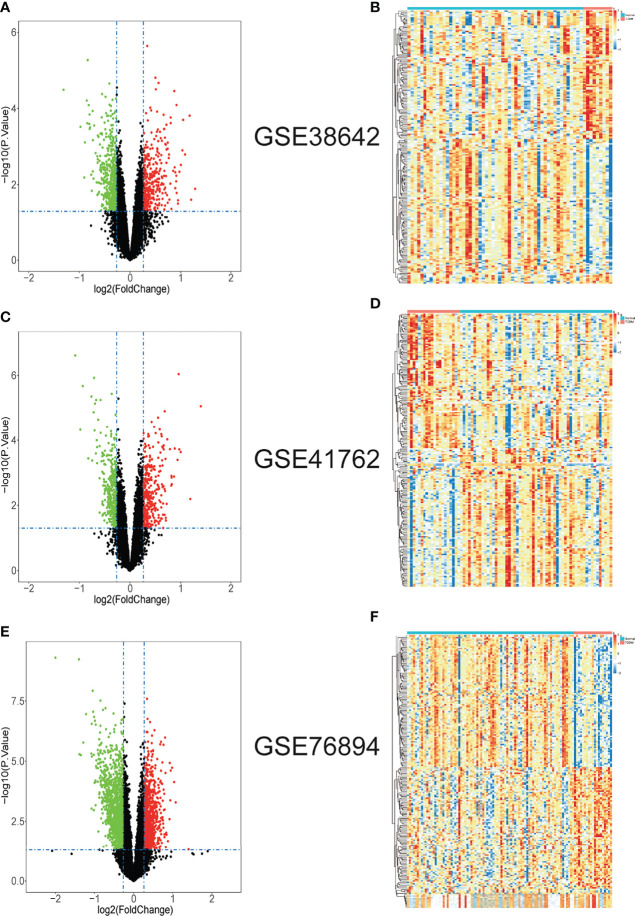
The gene differential expression in T2DM islet issues datasets. **(A)** The volcano plot of DEGs in GSE38642. **(B)** The heatmap of top 200 DEGs in GSE38642. **(C)** The volcano plot of DEGs in GSE41762. **(D)** The heatmap of top 200 DEGs in GSE41762. **(E)** The volcano plot of DEGs in GSE76894. **(F)** The heatmap of top 200 DEGs in GSE76894. In volcano plots, green dots represent down-regulated genes; red dots represent up-regulated genes; black dots represent not-statistically significant genes. In heatmap of top 200 DEGs, red blocks represent up-regulated genes; blue blocks represent down-regulated genes. The differences are set as |log_2_FC| > log_2_1.2 and *p* < 0.05.

**Figure 2:**
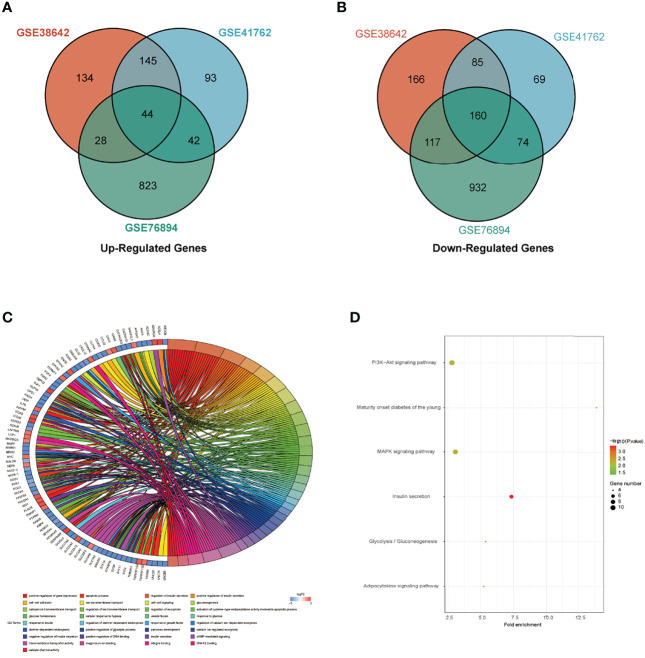
Identification of DEGs and functional enrichment analysis. **(A)** The intersection of up-regulated DEGs in GSE38642, GSE41762 and GSE76894 datasets. **(B)** The intersection of down-regulated DEGs in GSE38642, GSE41762 and GSE76894 datasets. **(C)** Biological process (BP) and Molecular function (MF) in all DEGs. **(D)** Kyoto encyclopedia of genes and genomes (KEGG) in all DEGs.

Three datasets GSE38642, GSE41762 and GSE76894 were analyzed by “limma” package in R with *p <*0.05 and |log_2_FC| >log_2_1.2 as the threshold. In dataset GSE38642, we isolated 879 DEGs, which contains 351 up-regulated genes and 528 down-regulated genes (**Figure 1A**). In dataset GSE41762, we extracted 712 DEGs, which contains 324 up-regulated genes and 388 down-regulated genes (**Figure 1C**). 2220 DEGs, comprised of 937 up-regulated genes and 1283 down-regulated genes were identified in dataset GSE76894 (**Figure 1E**). The top 200 DEGs in these datasets were respectively performed clustering and shown in the form of heatmaps (**Figures 1B, D, F**). 44 up-regulated DEGs and 160 down-regulated DEGs from intersection of three datasets was chosen by online tool Hiplot (https://hiplot.com.cn/) and shown in Venn diagram (**Figures 2A, B**). The percentages and gene names of DEGs in each dataset were shown in **Supplementary Material Table 1**.

### Results of Functional Enrichment Analysis

DEGs between T2DM and Normal islet tissues were mainly enriched in the following BP terms based on enrichment analysis: gene expression, apoptosis, insulin secretion, cell-cell adhesion, gluconeogenesis, ion transmembrane transport, exocytosis, cellular response to hypoxia. MF of DEGs were primarily enriched for transmembrane transporter activity, magnesium ion binding, integrin binding, SNARE binding, calcium channel activity (**Figure 2C**). KEGG analysis shown that DEGs were mainly participated in PI3K-Akt signaling pathway, MAPK signaling pathway and insulin secretion (**Figure 2D**). The concrete GO and KEGG terms of DEGs in each dataset were shown in **Supplementary Material Table 2**.

### Construction of Co-Expression Module and Identification of Key Trait-Module Genes

**Figure 3:**
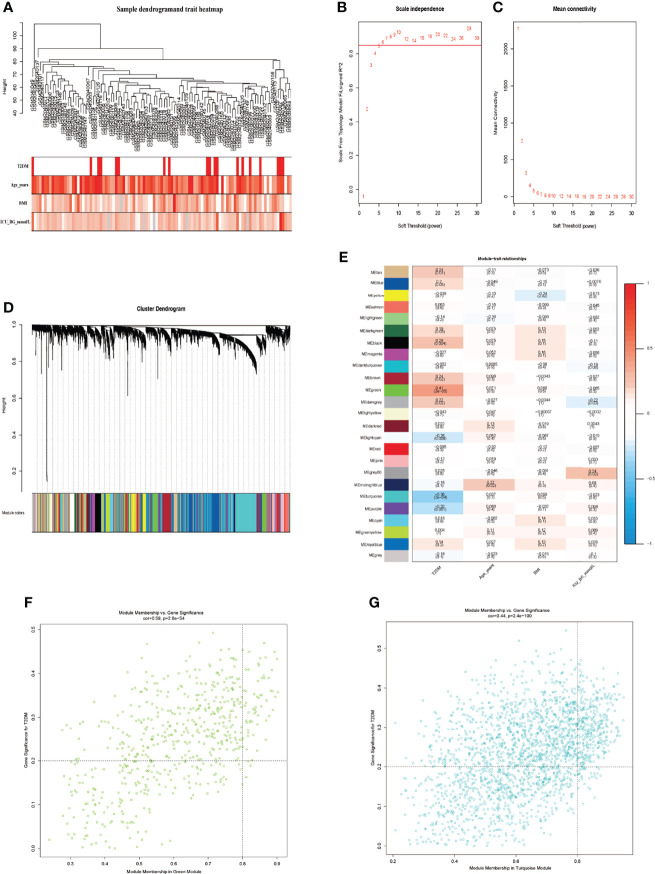
Screening of key trait-module genes using WGCNA. **(A)** Sample clustering tree and phenotypic heatmap. **(B)** The analysis of scale-free fit index and various soft-threshold powers. **(C)** The analysis of mean connectivity and soft-threshold powers. **(D)** Dendrogram clustered using Dynamic Tree Cut algorithm. **(E)** The relationships of different modules and traits. **(F)** The analysis of green module genes and T2DM. **(G)** The analysis of turquoise module genes and T2DM.

WGCNA, a good method to find the relationship of genes and traits, was performed to excavate key genes significantly related to traits by combining with DEGs. Sample clustering tree and phenotypic heatmap were shown in **Figure 3A**. The darker the color, the higher the expression in phenotypic heatmap, and the grey represented missing values. No sample was removed in dataset GSE76894 for no outlier. The top 75% genes of the median absolute deviation (MAD) in this dataset were screened for WGCNA. When the soft-thresholding power was equal to 6, the evaluation parameter *R* ^2^ was greater than 0.85 (**Figures 3B, C**). A scale-free network was constructed with the soft-thresholding power of *β* = 6 and the genes were classified into different modules according to expression profiles. We identified a total of 24 modules *via* the average linkage clustering (**Figure 3D**). Grey indicated genes that had not been classified into modules. Green module and turquoise module had the most significant gene significance (GS) in T2DM and there was no conspicuous module in other traits. (**Figure 3E**). Finally, we respectively extracted 73 genes in green module and 397 genes in turquoise module with MM >0.8 and GS >0.2 (**Figures 3F, G**, **Supplementary Material Table 3**). These 470 genes were considered as the trait-module key genes.

### Screening and Validation of T2DM-FRGs

**Figure 4:**
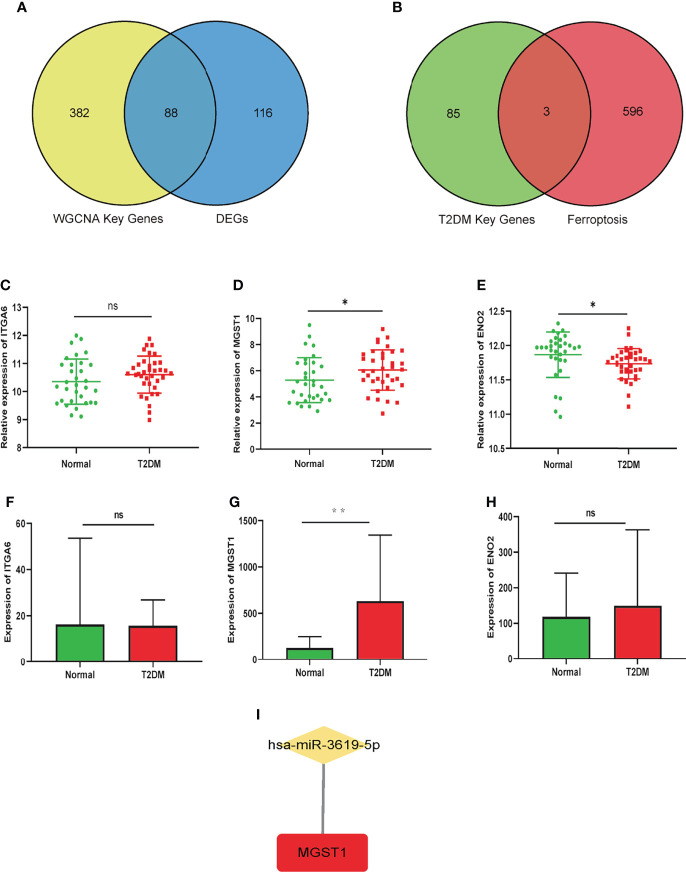
Screening of T2DM-FRGs hub genes and prediction of miRNA in T2DM and Normal groups. **(A)** The intersection of all DEGs and key module-trait genes in WGCNA. **(B)** The intersection of T2DM key genes and ferroptosis-related genes. Relative expression of ITGA6 **(C)**, MGST1 **(D)** and ENO2 **(E)** in GSE76895 dataset. Expression of ITGA6 **(F)**, MGST1 **(G)** and ENO2 **(H)** in E-MTAB-5061 dataset. Unpaired t-test, ns p> 0.05, *p< 0.05, **p< 0.01. **(I)** The prediction of miRNA regulated MGST1.

88 genes were elected from the union set between DEGs and trait-module key genes in WGCNA, which was visualized in Venn diagram (**Figure 4A**). These genes were intersected with ferroptosis-related genes contained 599 genes subsequently, and we obtained 3 T2DM-FRGs *(ITGA6*, *MGST1* and *ENO2*) eventually, shown in **Figure 4B**. *MGST1* and *ITGA6* increased in T2DM group, while *ENO2* decreased. GSE76895 and E-MTAB-5061 datasets were used to verified T2DM-FRGs. In single-cell sequencing dataset E-MTAB-5061, we selected the β cell samples to perform validation. The differential expression of *MGST1*, not *ITGA6* and *ENO2*, was proved to be statistically significant in two validation datasets (**Figures 4C–H**).

### Prediction of miRNA That Regulated Hub Gene

As shown in **Figure 4I**, has-miR-3619-5p may regulated *MGST1* by prediction in Targetscan (http://www.targetscan.org/vert_71/), TarBase v.8 (http://www.microrna.gr/tarbase), miRDB (http://mirdb.org/) and ENCORI (https://starbase.sysu.edu.cn/) miRNA databases.

## Discussion

IBCD is considered as the important factor in the progression of T2DM and is also another direction to treat T2DM. Long-term hyperglycemia, hyperlipidemia and uncontrol of blood glucose in patients with T2DM caused the islet β cells damage and even death (4). Some ways of islet β cells death in T2DM, such as apoptosis and autophagy were confirmed in studies (22, 23). But the early diagnosis and treatment of IBCD in T2DM is still a difficult and unsolved problem. As a newly identified iron-dependent form of oxidative cell death, ferroptosis remains unclear in T2DM. The features that type 2 diabetic islet β cells appear low antioxidant activity and iron overload indicated us the possible link between IBCD and ferroptosis (7, 9). We hope to discover the association between the two and detect the target for early diagnosis and treatment in IBCD of T2DM by our study.

MAPK and PI3K-Akt signaling pathways, which have been proved as the pathways regulated ferroptosis, were significantly enriched in KEGG of DEGs in IBCD of T2DM (24, 25). However, no significant ferroptosis-related terms were found in GO of DEGs. We considered that serious ferroptosis may have not occurred in IBCD at pre-middle stage of T2DM, but changes in ferroptosis-related pathways, such as MAPK and PI3K pathways. The differential genes between T2DM and Normal islet tissues were screened by combining DEGs and trait-related genes in WGCNA, which ensured the reliability of data. We obtained 3 T2DM-FRGs (*ITGA6*, *MGST1* and *ENO2*) from the intersection between differential genes and ferroptosis-related genes. We found *MGST1* and *ITGA6* is up-regulated, while *ENO2* is down-regulated in T2DM. *MGST1*, not *ITGA6* and *ENO2*, was considered as the key T2DM-FRG after validation in another type 2 diabetic islet issues dataset and single-cell sequencing dataset. This study mays provide reference for research the connection between IBCD and ferroptosis in T2DM.

*MGST1* (Microsomal Glutathione S-transferase 1) is a membrane-bound transferase regulates oxidative stress. It mainly located in mitochondria, ER plasma membrane and peroxisome (26). Researchers found that it fighted ferroptosis in pancreatic cancer cells. *MGST1* is regulated by Nrf2 (Nuclear Factor erythroid 2-Related Factor 2) and partly combined with ALOX5 (Arachidonate Lipoxygenases 5) to inhibit ferroptosis (27). In addition, *MGST1* is up-regulated during ferroptosis (27). We prove that mRNA expression of *MGST1* increases in type 2 diabetic IBCD. Thus, it is conceivable to hypothesis that *MGST1* mays contribute to alleviate ferroptosis in type 2 diabetic IBCD, which needs to be proved experimentally in the future. *ENO2* (Enolase 2 or gamma enolase, also known as neuron-specific enolase, NSE), is a glycolytic enzyme which catalyzes 2-phosphoglycerate to form PEP (Phosphoenolpyruvate). It not only participates in glycolysis and gluconeogenesis, but also is related with inflammation and ferroptosis (28, 29). *ENO2* mays influence ferroptosis by neuroglobin (29). A study indicates that *ENO2* is positively correlated with insulin secretion and negatively correlated with HbA1c (30). *ENO2* is decreased in cultured INS-1 cells treated with high glucose in 12 hours and in type 2 diabetic islet cells (30). These results *in vivo* and *in vitro* suggest that *ENO2* is a key molecule protect the function of islet β cells. However, mRNA expression of *ENO2* did not change in islet single-cell RNA sequencing samples. This may be related to the small single-cell sequencing sample size. Whether *ENO2* regulates islet β cells in T2DM through ferroptosis pathway remains to be studied.

In addition, the study about islet issue sequencing has certain limitations. There is no way to know whether the target gene changes are present in β cells because islet issues include a variety of cells, such as α cells, β cells and PP cells. There are many studies about islet single-cell RNA sequencing of T2DM at present (16, 31, 32). Therefore, we used the islet single-cell sequencing dataset to check the target genes again. Single-cell sequencing, though, is more specific to get a lot of β cells and its sequencing data, but contains a few samples in patients for its high cost, which will cause the inability to exclude individual differences. Finally, we selected *MGST1* as the potential ferroptosis-related gene in IBCD of T2DM by combing large patient samples of islet issue sequencing datasets and specificity of islet single-cell sequencing dataset.

This study reveals the potential relationship between ferroptosis and IBCD in T2DM based on bioinformatics analysis. It deeply adds to our understanding of type 2 diabetic islet β cells impairment and provides reference for developing targeted therapy for T2DM.

## Data Availability Statement

The original contributions presented in the study are included in the article/**Supplementary Material**. Further inquiries can be directed to the corresponding authors.

## Author Contributions

HY designed the study and wrote manuscript, HY and RW conducted the bioinformatic analysis. JW and YW took part in a discussion. XZ and LW amended the manuscript and co-wrote the final draft with HY. All authors contributed to the article and approved the submitted version.

## Funding

This study was supported by Talent introduction funding project of Guangzhou Overseas Chinese Hospital, Jinan University (no.808026).

## Conflict of Interest

The authors declare that the research was conducted in the absence of any commercial or financial relationships that could be construed as a potential conflict of interest.

## Publisher’s Note

All claims expressed in this article are solely those of the authors and do not necessarily represent those of their affiliated organizations, or those of the publisher, the editors and the reviewers. Any product that may be evaluated in this article, or claim that may be made by its manufacturer, is not guaranteed or endorsed by the publisher.
